# Recovery in schizophrenia: the role of antipsychotic treatment

**DOI:** 10.1192/j.eurpsy.2024.64

**Published:** 2024-08-27

**Authors:** I. Bitter

**Affiliations:** Psychiatry and Psychotherapy, Semmelweis University, Budapest, Hungary

## Abstract

**Introduction:**

Comprehensive care programs, which include individually planned pharmacotherapy are associated with higher rates of recovery^1^ and better long-term prognosis^2^. However, there are barriers to individually optimised antipsychotic treatment both from both the patients and treatment teams perspectives.

**Objectives:**

To summarize the potential contribution of adequate long-term antipsychotic treatment to recovery or better outcomes in schizophrenia.

**Method:**

Review of research data.

Results A shorter duration of untreated psychosis, a lower number of relapses, and the absence of a chronic course of psychosis are associated with higher rates of recovery and a better prognosis. The OPUS early intervention program was associated with better outcomes for up to 10 years, but not for more than 20 years^3^. Second generation antipsychotics are associated with lower mortality rates, including suicides in young people with schizophrenia.^4^

Higher doses of antipsychotics are associated with poorer outcomes and with potential structural brain changes, while adequate (lower) doses of antipsychotics are associated with lower side effect burden and better overall outcomes^5^. A significant proportion of patient may benefit from polypharmacy (combination of 2 antipsychotics)^6^. Antipsychotic treatment discontinuation strategies are associated with the development of treatment resistance.^7^

**Conclusions:**

Adequate (low dose) antipsychotic treatment is part of the complex early intervention programs and long term treatment of schizophrenia, which are associated with higher rates of recovery and good outcomes. The role of polypharmacy (combination of 2 antipsychotics) may need a reconsideration in the treatment guidelines of schizophrenia.

1. Kane JM et al. The Journal of clinical psychiatry. 2015 Mar 25;76(3):16590.
2. Posselt CM et al. 2021. American Journal of Psychiatry, 2021, 178(10):941-951
3. Hansen HG et al. 2023. JAMA Psychiatry, 2023, 80(4): 371-379.
4. Correll CU et al. World Psychiatry. 2022 Jun;21(2):248-71.
5. Andreasen NC et al. 2013 Jun;170(6):609-15.
6. Katona L et al. 2014 Jan 1;152(1):246-54.
7. Emsley R et al2013 Feb 1;33(1):80-3.

**Disclosure of Interest:**

None Declared

